# A Clearer Picture of China’s Air: Using Satellite Data and Ground Monitoring to Estimate PM_2.5_ over Time

**DOI:** 10.1289/ehp.124-A38

**Published:** 2016-02-01

**Authors:** Nate Seltenrich

**Affiliations:** Nate Seltenrich covers science and the environment from Petaluma, CA. His work has appeared in *High Country News*, *Sierra*, *Yale Environment 360*, *Earth Island Journal*, and other regional and national publications.

In early December 2015 Beijing made the international news for its hazardous, heavily polluted air, culminating with the city’s first-ever air-pollution “red alert,” a designation that closed schools and strictly limited vehicle traffic for three days.^1^ A similar scare occurred in January 2013, when hourly readings of fine particulate matter (PM_2.5_) levels in the Chinese capital peaked at 886 µg/m^3^.^2^ (By comparison, the World Health Organization recommends that PM_2.5_ averaged over 24 hours not exceed 25 µg/m^3^.^3^) Yet despite the severity of these headline-grabbing episodes, average PM_2.5_ levels in Beijing and China overall appear to have decreased since approximately 2008, according to a study reported in this issue of *EHP*.^4^

The absence of a nationwide ground-monitoring network in China has limited researchers’ ability to assess the extent of pollution beyond major cities, says coauthor Yang Liu, an associate professor of environmental health at Emory University. It has also hampered research on the adverse impacts of chronic exposure to PM_2.5_, one of the air pollutants most consistently associated with human health effects.^5^

**Figure:**
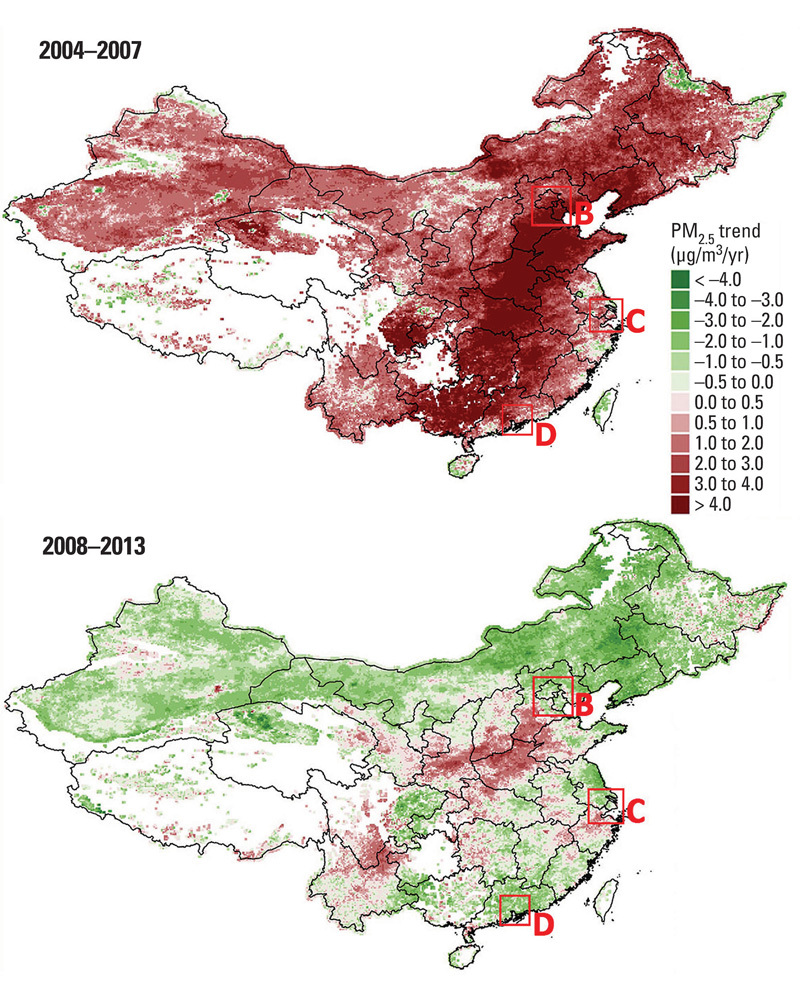
Estimates of average annual increases or decreases in PM_2.5_ during two time periods show a general declining trend in air pollution across China—even in the densely populated Beijing-Tianjin metropolitan region (B), Yangtze River delta (C), and Pearl River delta (D)—although in some areas concentrations continued to increase after 2008. White areas indicate missing data. Ma et al. (2016)^4^

Other research teams have experimented with estimating ground-level PM_2.5_ levels from satellite readings of a measurement known as aerosol optical depth (AOD), using statistical models to fill data gaps left by ground stations.^6^^,^^7^^,^^8^^,^^9^ However, this approach requires a sufficient ground-monitoring network to validate the satellite-based models.

New possibilities for PM_2.5_ exposure estimates and epidemiological research in China opened up in late December 2012, when the government began a rapid and extensive rollout of ground-monitoring stations nationwide, Liu says. He and his colleagues capitalized on this network and demonstrated the combined power of ground monitoring and remote sensing by integrating 10 years of AOD data with ground-based air quality measurements taken by 1,185 ground monitors distributed throughout the country. The researchers used data from 2013 and the first six months of 2014, the period during which the two measurement sources overlapped, as the basis for a model they used to extrapolate back through January 2004. The result was a decade’s worth of estimated daily, monthly, and seasonal concentrations of ground-level PM_2.5_ nationwide.^4^

Relevant to the recent news on Beijing’s air-quality crisis, the researchers also present results that undermine the prevailing media narrative that pollution is only getting worse in China. They found that while PM_2.5_ levels averaged nationwide—and in the Beijing–Tianjin metropolitan region specifically—were indeed slightly higher in 2013 than in 2004, they had been steadily declining since approximately 2008. Still, the trend wasn’t universal: Industrial and rapidly developing regions southwest of Beijing and in South Central China were the exception to the rule, with pollution levels increasing throughout the entire period.^4^

“These historical estimates allow us to look at the health effects of longer-term exposure,” says Michael Brauer, a professor of public health at the University of British Columbia, who was not involved with the study. He explains that long-term exposures are most important in estimating public health impacts, such as how air pollution exposures affect disease development and premature death. “With these kinds of exposure estimates, we can link to existing data sets and studies looking at all kinds of things to understand the development of cardiovascular disease, cancer, and more,” Brauer says.

The new findings are largely corroborated by a book on the subject published in 2013 by the Harvard China Project, says project executive director and co-editor Chris Nielsen.^10^ “The inflection point in 2007 and 2008 is very consistent with what we’ve concluded by looking at the effects and timing of emission controls and other factors on air quality,” he says. These factors include policies addressing energy intensity, industrial emissions, and vehicle emissions, as well as broader economic trends, the 2008 Beijing Olympics, and, potentially, meteorological factors tied to climate change.

Nielsen, who was not involved with the current study, points out that discrete episodes of severe PM_2.5_ are a related but separate phenomenon from annual average PM_2.5_. The trends for each can go in opposite directions, he says, with episodes getting somewhat worse while annual averages get somewhat better.

Liu describes his team’s model as a data-driven approach to expand the reach of China’s newly enhanced ground-monitoring network. “With the satellite data we are able to finally reach out to the suburban and rural communities,” he says. “Historically they have been left out of these sorts of studies.”
